# The intervention of local public authorities and the impact of the COVID-19 pandemic in Romania: a subnational analysis

**DOI:** 10.3389/fpubh.2024.1105518

**Published:** 2024-05-16

**Authors:** George Ștefan, Dumitru Alexandru Bodislav, Alina Arsăni (Chiriță), Andrei Hrebenciuc, Ada Paierele, Anca Paraschiv, Daniela Virjan

**Affiliations:** Department of Economics and Economic Policies, Bucharest University of Economic Studies, Bucharest, Romania

**Keywords:** COVID-19 pandemic, investment, local public authorities, cluster analysis, subnational administration

## Abstract

The COVID-19 pandemic had a strong territorial dimension, with a highly asymmetric impact among Romanian counties, depending on pre-existing vulnerabilities, regions’ economic structure, exposure to global value chains, specialization, and overall ability to shift a large share of employees to remote working. The aim of this paper is to assess the role of Romanian local authorities during this unprecedented global medical emergency by capturing the changes of public spending at the local level between 2010 and 2021 and amid the COVID-19 pandemic, and to identify clusters of Romanian counties that shared similar characteristics in this period, using a panel data quantitative model and hierarchical cluster analysis. Our empirical analysis shows that between 2010-2021, the impact of social assistance expenditures was higher than public investment (capital spending and EU funds) on the GDP per capita at county level. Additionally, based on various macroeconomic and structural indicators (health, labour market performance, economic development, entrepreneurship, and both local public revenues and several types of expenditures), we determined seven clusters of counties. The research contributes to the discussion regarding the increase of economic resilience but also to the evidence-based public policies implementation at local level.

## 1 Introduction

The COVID-19 pandemic, which quickly spread around the world, has had a high impact on both central and local governance, with the outbreak in February – March 2020, leading to the Great Lockdown in April (1). Considering the unprecedented scale of the COVID-19 crisis, comparisons with recent crises, including the 2008–2009 financial crisis, have significant limitations (2). The impact on both the supply side and demand side, as well as on all sectors and regions of the world proved that the COVID-19 crisis was unique.

Pandemics are a fairly rare type of natural disaster, which also includes extreme weather events and geological disasters. Historically, there have been at least 15 large pandemics causing more than 100,000 deaths. Apart from the plague in the Middle Ages (1331–1353) causing 75 million deaths, the Spanish flu (1918–1919) was the most devastating pandemic in recent history with 100 million deaths (3, 4). In more recent times, the Hong Kong flu (1968–1969), the Asian flu (1957–1958), SARS (2002–2003), swine flu (2009–2010) and, MERS (2012–2013), Ebola (2014–2015), Zika (2016) had also caused significant impact on lives and livelihoods, but the total number of deaths was more limited. However, COVID-19 differs from previous pandemics in the breadth of coverage in terms of the number of countries and people at risk (5).

The central and local authorities faced tough trade-offs between mitigating the impact on lives and livelihoods and managing the economic recovery, because, beyond the spread of the pandemic, further challenges started to emerge such as reduced demand, supply chain shocks, rising unemployment, especially in sectors such as accommodation and services and transport, and inflation, with little room for maneuver in terms of fiscal revenues.

To limit the spread of the virus, authorities had introduced major containment measures, while for managing the economic recovery strong fiscal and monetary and macro-financial responses were put in place (6). However, the COVID-19 pandemic and associated confinement measures had pushed the European economy, including Romania, into a sudden recession, with the deepest output contraction since World War II (7).

Ex-ante, Romania was considered less resilient due to substantial economic and social disparities among Romanian regions (41 counties plus the municipality of Bucharest) in terms of standard of living, access to education, information and technology, access to health, regional administrative capacity (8), level of income and purchasing power (9), and entrepreneurial environment (10). However, according to the Fitch Rating Agency, Romanian municipalities proved to be more resilient to the pandemic than initially expected (11).

Romania has a mix of financial instruments aimed at building fiscal resilience against disasters, including: (i) an annual budget line (ii) a contingent fund, and (iii) a World Bank Development Policy Loan with a catastrophe deferred drawdown option (Cat DDO), a contingent financing line which was signed in 2018 (12, 13). Nevertheless, given the devastating economic and social disruptions caused by the pandemic, local authorities were forced to allocate higher financial resources from their budgets to support the economy: investments or incurred expenses related to the management and development of the spaces necessary to treat COVID-19 cases (new centers and spaces for testing and treatment, proper equipment and machines, masks, coveralls, disinfectants, and other auxiliary materials), personnel expenses (medical and auxiliary staff, and in some counties the increase was over 50%), or social expenses for supporting people with reduced income or unemployed. Local authorities are strongly affected by the economic policies created and implemented by the central authorities. Thus, in this macroeconomic context impacted by the pandemic and military conflict, the authorities, not only in Romania, considered that a sudden stop of the economy would have a much greater negative impact than the management and cost of the fiscal deficits.

The aim of this paper is to assess the role of Romanian local authorities during the COVID-19 pandemic, by spotting patterns in terms of public spending between 2010 and 2021 in areas related to health and non-health issues, as well as to identify several clusters of counties with similar socio-economic characteristics. According to our knowledge, this type of scientific research for Romania has not been done before by other researchers and thus our inquiry is completely original in the economic research landscape in Romania and contributes to the literature that deals with the understanding of the role and intervention of public authorities amid the COVID-19 pandemic.

The remainder of the paper is structured as follows. The second section provides a review of the literature regarding the pandemic effects and measures implemented by public authorities, to tame the negative impact on economic activity. The third part of the article analyzes the Romanian context amid the COVID-19 pandemic and its economic consequences.

The fourth section covers empirical analysis. The methodology is based on a panel data model applied at the county level (42 counties) assessing the impact of two types of local public expenditures (public investment and social assistance expenditures) between 2010 and 2021 on GDP per capita among Romanian counties. Furthermore, based on various macroeconomic indicators and using a Hierarchical Cluster Analysis, we generated different clusters of counties based on their structural characteristics (development level, health, public expenditures and revenues at the local level, labor market, and entrepreneurship). The last section includes conclusions and further research proposals based on our results. The annexes include the authors’ own processing using the SPSS26 program and several figures and tables used in data research.

## 2 Literature review

It is well known that pandemics had seriously worsened the economic situation in affected regions. In general, pandemics are characterized by: (i) a contraction of business activity; (ii) a reduction of consumer demand due to high uncertainty; (iii) a deterioration of the labor market: jobs losses due to the narrowing of consumer demand and contraction of production; a reduction in the labor supply due to morbidity and mortality, reduced mobility; (iv) a hike in the cost of doing business due to the need to comply with sanitary and hygienic requirements; (v) a disruption on trade relations, the impossibility of organizing optimal logistics; (vi) a change in the structure of demand due to social distancing, and (vii) a decrease in investment due to uncertainty in the medium and long term.

Eichenbaum et al. (14) underline the need to find a balance between recessions caused by epidemics and economic measures to contain them. The COVID-19 pandemic was alarming as it had several unfamiliar features: the outbreak was considered a natural disaster, while the development was predetermined by the targeted actions of the governments of almost all countries to contain the spread of the virus by squeezing business activity and limiting population migration. Given the unique economic shock, the coordination of monetary and fiscal policy aimed at supporting the living standards of the population, curbing the decline in employment and investment, and preventing bankruptcies (15).

The COVID-19 crisis happened within an integrated global economy, spanning many trade and financial networks, thus, the adverse impact of the pandemic was multiplied many times (15). Central and local public authorities implemented important financial and economic measures aimed at overcoming recessions, mitigating their course, and taming the consequences for the population and business.

Literature shows that structural factors and the rapid reaction of the authorities had an important role in mitigating COVID-19. Based on an Artificial Neural Network that analyzed data from 192 countries to explain the COVID-19 impact on the number of deaths and the fatality rate at the national level, Magazzino et al. (16) shows that using different vaccination plans and campaigns, the national turnout in the participation at the vaccination campaign, and the number of doses administered, countries under study reduced the fatality rate of COVID-19, and the fatality rate collapses with increasing doses administered.

Additionally, Magazzino et al. (17) show for the Hubei region (China) that the initial conditions of pollution and fossil fuel use are important. For better control of COVID-19 diffusion, the authors highlight the importance of air quality enforcement (especially in terms of pollutant particles such as PM2.5, PM10, and CO_2_) to minimize public health loss and other diseases (heart and lung disease). See also the work of Mele et al. (18) regarding the role of NO2 concentration in Paris, Lyon, and Marseille (France) in spreading the COVID-19 epidemic.

Also, one interesting perspective was analyzed by Kehr et al. (19). The authors empirically examine the relation between the behavioral compliance and acceptance of COVID-19 regulations imposed by local authorities in New York and psychologically features of the individuals (like death anxiety). See also the study of Dimitrijevska-Markoski et al. (20) on how the local government officials affect both the citizens COVID-19 risk perception and support for COVID-19 mitigation measures in United States. However, most countries and regions were underprepared due to several reasons (2): (i) underestimation of the risk when the outbreak emerged (ii) lack of crisis management plans (with the exception of Asian countries that faced the SARs pandemic and, for example, the Nordic countries known for their thorough crisis management plans) (iii) lack of basic equipment such as masks and (iv) reduced public expenditures and investments in health equipment and hospital infrastructure.

Thus, more granularly, all local administrations were forced to become relevant actors in managing the situation of crisis, being responsible for applying isolation measures, ensuring health services and social, but also economic growth and development. Urban areas were the ones that managed the health crisis best, becoming the epicenter of the pandemic measures, including in terms of budgetary planning of expenditures and revenues (21).

Usually, in Romania, in counties with a GDP per capita below the national average, the share of personnel spending in total expenditures is higher, on the background of scarce opportunities on the labor market amid the underdeveloped private sector, which makes the local public administration one of the main employers. Moreover, in these counties, there is a limited capacity for investments due to the lack of local public revenues (less economic activity) and the reduced ability to attract EU funds.

**Figure 1:**
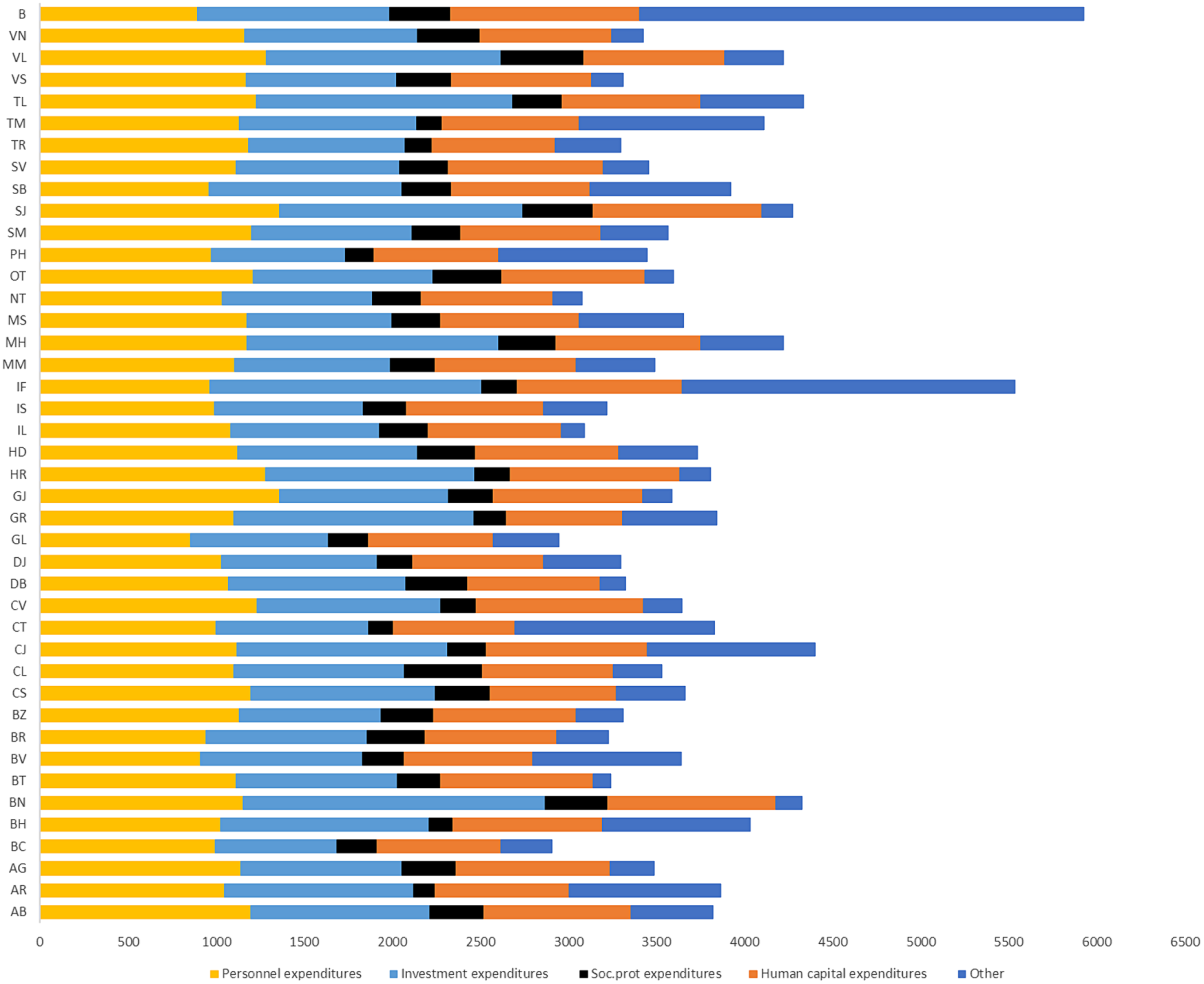
Local public spending categories (change 2019–2020). Source: authors’ calculation based on MDLPA and INS data.

Figure 1 presents the structure of public expenditures at the county level: (i) investment (EU funds and capital spending) (ii) human capital (education and health) (iii) social protection, and (iv) personnel spending. The values are expressed as RON per capita for each of the four categories. The results reveal that personnel spending is the most important category of expenditures, from 15% in Bucharest out of total spending to 37% in Gorj.

Also, amid the COVID-19 pandemic, local public expenditures changed significantly in 2020 compared to 2019, especially investment expenditures in different sectors. Our findings based on the data from the Ministry for Development, Public Works and Administration (MDLPA) show that 33 out of 42 counties increased the share of their investment expenditures in 2019–2020, while for other types around ¼ counties increased social protection, personnel, and health and education spending. In Brașov, Tulcea, and Bihor the share of investment in total local public expenditures increased by more than 10 percentage points (pp).

In terms of education and healthcare spending, major increases within the local public spending structure were registered in Harghita, Ilfov, and Sibiu, while in Vaslui, Brăila, and Argeș they decreased significantly. Moreover, correlated with the impact of COVID-19 (the share of COVID-19 deaths in total cases at the county level) Brăila, Argeș and Suceava were among the most affected counties.

**Figure 2:**
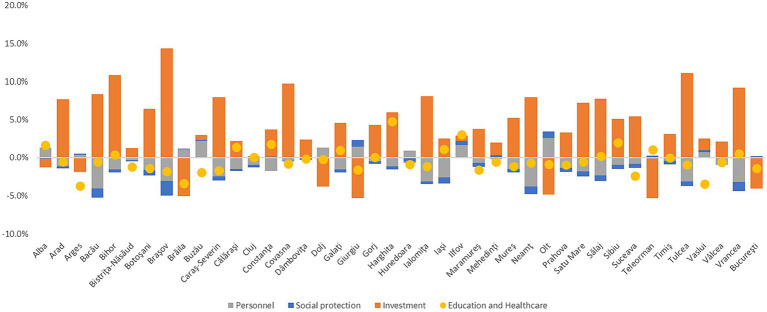
Investment/passive expenditures ratio (average 2010–2021). Source: authors’ calculation based on MDLPA data.

Furthermore, based on the county-level data from MDLPA, we computed the ratio of average EU funds and capital spending (*investment expenditures*) in the personnel and social assistance expenditures (we called them *passive expenditures*) during 2010–2021, in order to identify the main budgetary direction of local public authorities during this timeframe (see Figure 2).

According to our analysis, counties oriented toward investments were Bistrița-Năsăud, Ilfov, Bihor, and Giurgiu, reflecting higher medium value of investment expenditures per capita than personnel and social assistance exp.

While most of the studies looking at the socio-economic impact of the pandemic focus on the national / central level, only few have considered subnational levels. Thus, our study aims to address this gap in the literature since the papers analyzing the pandemic impact on Romanian NUTS 3 regions published so far cover only a limited number of counties and very specific sectors such as cultural and creative activities (22), tourism (23) or look rather at the budgetary pressure to cover health expenditure through developing a sustainability index for public health (24).

## 3 Romanian landscape amid COVID-19 pandemic

The consequences of the pandemic were felt unevenly in Romania due to the significant gaps among regions in terms of demographic potential, access to resources and investment, degree of qualification and specialization of the labor force, the degree of digitization and technology, but also the administrative capacity to absorb European funds. The differences are accentuated between urban and rural areas, where the workforce is aging, productivity is lower, the unemployment rate is increasing, the degree of poverty and social exclusion is higher, and the standard of living is below average.

In Romania, the COVID-19 pandemic has deepened existing inequalities for different minorities in Romania, a large number of households being affected by the negative effects of the GDP contraction. Food security in several low-income rural areas became a growing problem for the government, one of the main concerns being represented by the employment channel. The measures taken by the central authorities reduced the impact on the formal workers but disregarded the informal workers, who were left without social protection (25).

The marginal communities were hardly hit by the disruptions in the health sector, the difficulties in accessing adequate health protection materials being highlighted by several institutions during the State of Emergency. The education system was severely affected due to the lack of stable internet access and lacking proper equipment. The Romanian Institute of Statistics stated that around one million of children were impacted in 2020 and 2021, determining a long-lasting effect on their educational skills.

The COVID-19 pandemic severely impacted the labor market from three perspectives: (i) number of jobs (ii) quality of work and (iii) social protection access (26). The government decisions aimed to reduce social contacts led to a reduction of labor demand and a temporary increase of the unemployment rate.

**Figure 3:**
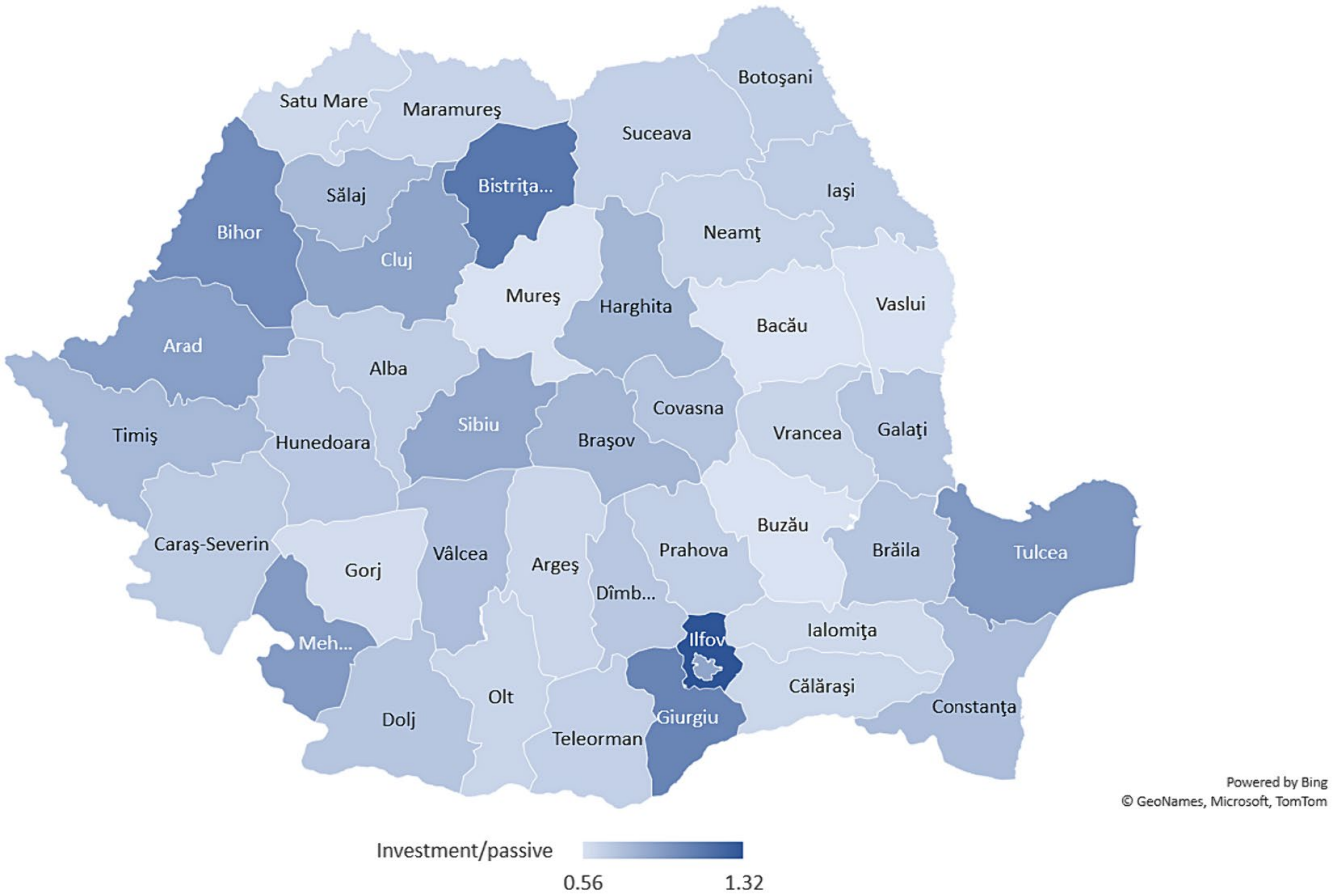
Nominal GDP per capita and total public expenditures dynamic (2019–2020). Source: authors’ calculation based on INS data.

Among Romanian counties the impact of COVID-19 shock was important, but the magnitude was different depending on the initial economic structure and the capacity of the public authorities to intervene. Thus, there was a high level of heterogeneity within Romanian regions, in seven counties the nominal GDP per capita actually increased, especially in Buzău, even though the local public intervention was modest in terms of expenditures to support the demand (see Figure 3). Also, in Giurgiu, Alba and Galați, the nominal GDP per capita stagnated, while it decreased in the others 32 counties.

**Figure 4:**
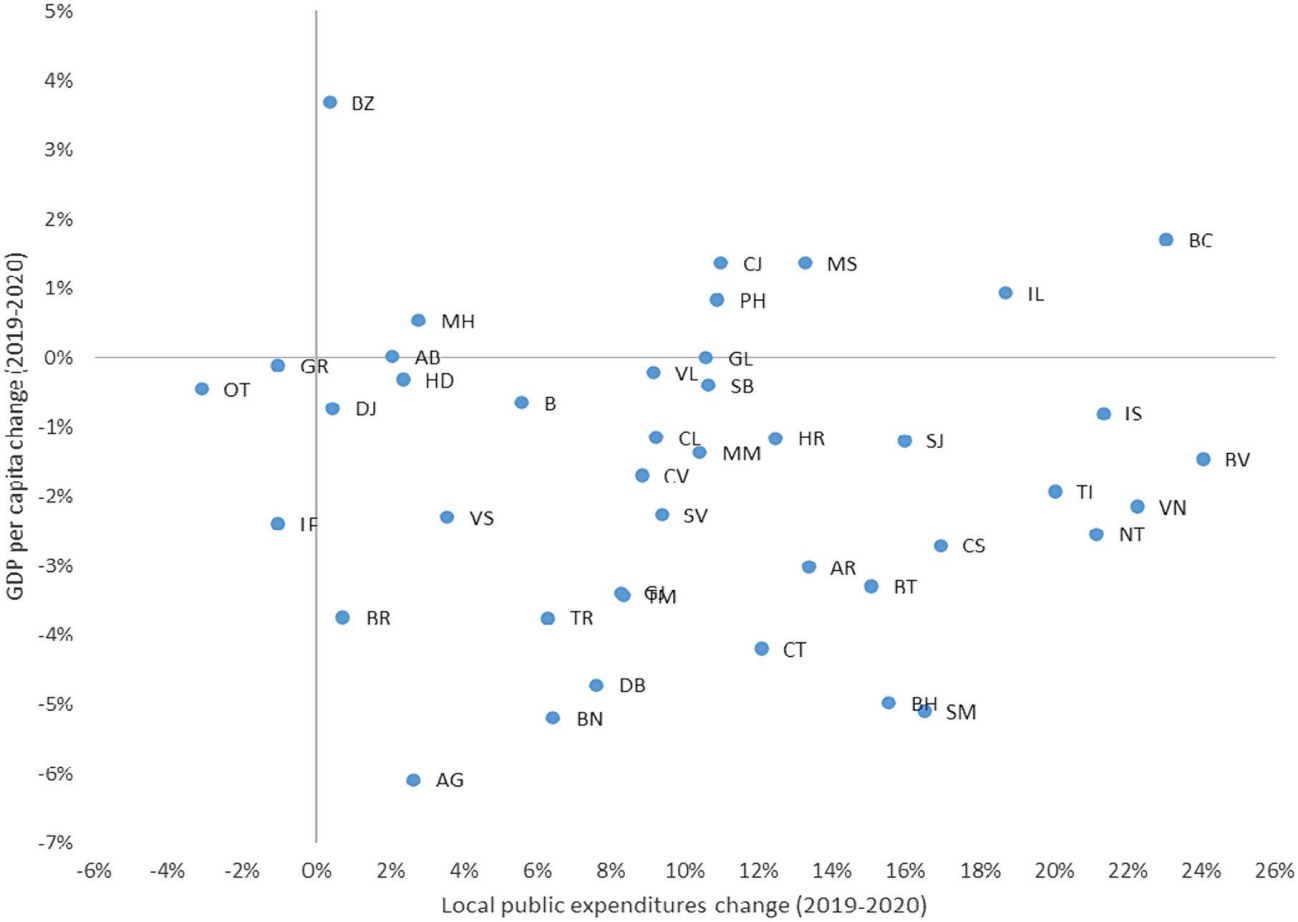
Structure of local public spending at county level (average 2010–2021, RON per capita). Source: authors’ calculation based on MDLPA data.

As highlighted in Figure 4, the GDP per capita registered a significant decline in Argeș, Bistrița Năsăud, Dâmbovița, Bihor and Satu Mare, even though the local public expenditures increased, with values larger than 5%. Important to observe is that the decline in GDP per capita was high in both more industrialized counties (with a large number of firms, employees and services, like Bistrița Năsăud, Timișoara, Arges, Constanța, Bihor) as well as in less developed counties (Teleorman, Botoșani, Dâmbovița), where agriculture is more important, and the number of firms – the economic activity, respectively, – is significantly lower.

## 4 Empirical analysis

Local and regional authorities (LRAs) have been at the forefront in responding to this crisis in the EU. The COVID-19 effects on the economy have imposed a significant strain on LRA finances. The Local and Regional Finance and aftermath of COVID-19 Report (27) emphasizes the simultaneous rise in expenditure for public health, social services, social benefits and support for businesses, workers and citizens which was accompanied by decreased revenue from a significant reduction in economic activity as well as tax relief and deferment, in all EU countries, in different percent of local public budgets.

The organization of sub-national government in Romania is based on 41 counties plus Bucharest at an intermediate level and 3,181 municipalities (divided into communes, towns, and cities) with different public spending structure (Figure 1). Unfortunately, the Romanian local and regional authorities are still highly dependent on transfers from central government (81.2%), even if the local financing became more decentralized over the last years. Their own revenues, 10.4% from taxes (mainly on property, land, and vehicles) and 6.7% tariffs and fees cover only a small part of their total financial needs. Sub-national governments also have debt ceilings. Local government spending, representing 23.5% of total public expenditure, is most concentrated, more than the EU average, on education, health, housing, and community amenities.

**Figure 5:**
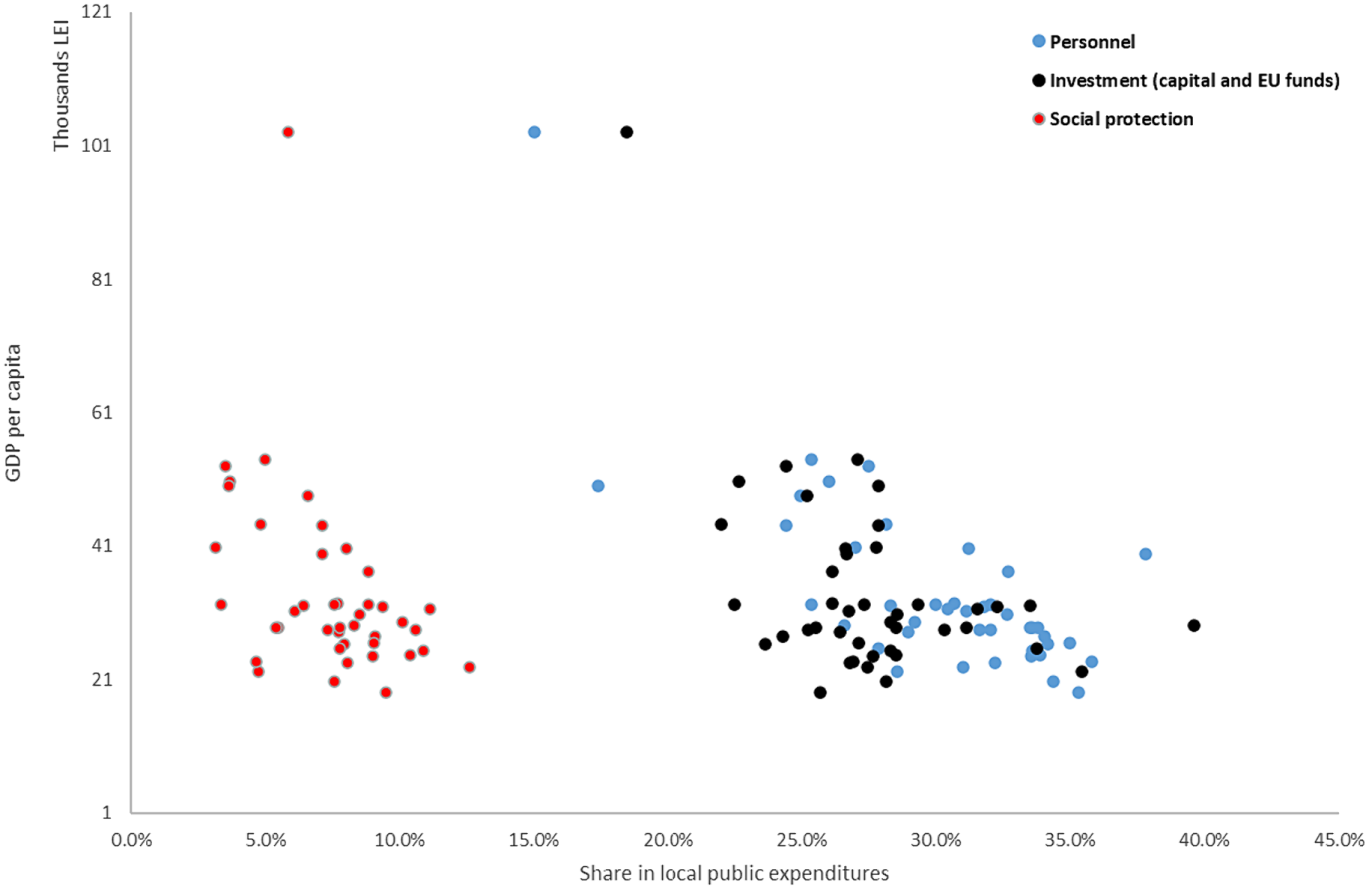
Relationship between GDP per capita and public spending categories. Source: authors’ calculation based on MDLPA and INS data.

It is important to note that among Romanian counties both personnel and investment (capital and EU funds) expenditures are highly negatively correlated with GDP per capita (see Figure 5). Even on a smaller scale, the situation is available also for social protection (social assistance) expenditures (see Figure 2).

## 5 Methodology – panel data analysis

To analyze the economic impact of local public expenditures, we used a panel data model where the dependent variable is the GDP per capita rate of change (GDP), and the independent variables are (i) public investments (capital expenditures and non-reimbursable European funds) (INVESTPUB) and (ii) social assistance expenditures (SOCIALPROT) from local budgets.

As presented in Table 1, we tested both expenditure categories that are traditionally considered to support economic growth, such as capital expenditure, but also those that rather have a short-term impact and increase current consumption, such as social assistance expenses (pensions, minimum income, unemployment benefits, etc.).

**Table 1:** The table should be after the Estimation Equation and Equation with Substituted Coefficients because it presents the results of the model.

Dependent Variable: LOG(D(GDP))				
Method: Panel Least Squares				
Date: 11/04/22 Time: 10:06				
Sample (adjusted): 2013 2021				
Periods included: 9				
Cross-sections included: 42				
Total panel (unbalanced) observations: 176				
Variable	Coefficient	Std. Error	t-Statistic	Prob.
C	0.662761	1.465825	0.452142	0.6517
LOG(D(INVESTPUB(−2)))	0.179962	0.052644	3.418459	0.0008
LOG(D(SOCIALPROT))	0.251616	0.069024	3.645360	0.0004
R-squared	0.122877	Mean dependent var		7.867152
Adjusted R-squared	0.112737	S.D. dependent var		0.897250
S.E. of regression	0.845161	Akaike info criterion		2.518319
Sum squared resid	123.5734	Schwarz criterion		2.572362
Log likelihood	−218.6121	Hannan-Quinn criter.		2.540238
F-statistic	12.11789	Durbin-Watson stat		2.072987
Prob(F-statistic)	0.000012			

Source: authors’ prelucration based on Eviews 8 program.

Both explanatory variables were expressed per capita, considering the county’s population by residence on July 1. We used annual data, covering the period 2010–2021. Datasets used in analysis were provided by Ministry for Development, Public Works and Administration^1^, Romanian National Statistics Institute (Tempo Database^2^) and National Commission for Strategy and Prognosis^3^. Also these are the data sources for the cluster analysis presented in the next section, alongside the Romanian National Institute for Public Healthcare.

**Estimation Equation:**


$$\begin{array}{l} \mathrm{LOG}\left(D\left(GDP\right)\right)=C\left(1\right)+C\left(2\right)*\mathrm{LOG}\left(D\left(\mathrm{INVESTPUB}\left(-2\right)\right)\right)\\ +C\left(3\right)*\mathrm{LOG}\left(D\left(\mathrm{SOCIALPROT}\right)\right) \end{array}$$

**Substituted Coefficients:**


$$\begin{array}{l} \mathrm{LOG}\left(D\left(GDP\right)\right)=0.662+0.1799^{*}\mathrm{LOG}\left(D\left(\mathrm{INVESTPUB}\left(-2\right)\right)\right)\\ +0.2516^{*}\mathrm{LOG}\left(D\left(\mathrm{SOCIALPROT}\right)\right) \end{array}$$

For investment expenses, we used the hypothesis that their impact on the GDP per capita is not simultaneously manifested, but with a delay (*lag*) of 2 years. The reasoning behind is the consistency of the *coefficients* obtained, and the fact that, in general, investment projects, mainly greenfield ones, are made on a longer term, the first year reflecting the design phase, thus local capital expenditure inflows are limited.

The results of running the model for the county-level analysis between 2010 and 2021^4^ highlight that social spending had a higher influence on the GDP per capita compared to investment expenses. Table 1 shows that the model is methodologically valid as both coefficients have a confidence level larger than 95%, while the errors autocorrelation is absent as the Durbin-Watson coefficient is close to 2. However, the low value of R-squared indicates that our two explanatory variables have a lower power to explain the entire evolution of the GDP per capita and there should be used more variables for better representativeness of the model.

The model shows that a 1% increase in social assistance spending leads to a simultaneous increase in GDP per capita by 0.25 p.p. while an increase in investment spending in the previous period (t-2 years) led to an increase in GDP per capita in the present with a value of 0.18 p.p.

Behind these results stands the high regional disparities between the capital city, Bucharest, alongside the more developed five counties^5^ and the rest of the 37 countries. This increases the sensitivity of local economies relative to changes in consumption, usually financed by wages and social protection benefits. Moreover, poorer counties have a higher consumption marginal propensity and thus the multiplier of social expenditures could be higher with a direct impact on local GDP per capita, strengthening the argument above.

Furthermore, large investment expenses are usually concentrated in large cities – where the accumulation of capital is already at a high level -, therefore, the impact of a marginal increase in investment stock generates a smaller stimulus in terms of GDP increases. However, further research is needed in order to obtain a more accurate image regarding the pass-through mechanism between local public investment, social protection benefits, and GDP per capita increase.

Even though the coefficients of the model are statistically significant and consistent, the R-squared has a low value, around 12%. The explanatory power of the selected independent variables is small because there are also other important factors behind the GDP per capita change during this period – private salaries, foreign investment, local competitiveness and exports, fiscal stimulus and state aid for development from central government, credit conditions, other economic sectors dynamic (construction, manufacture, agriculture in some counties) and others.

## 6 Cluster analysis

**Figure 6:**
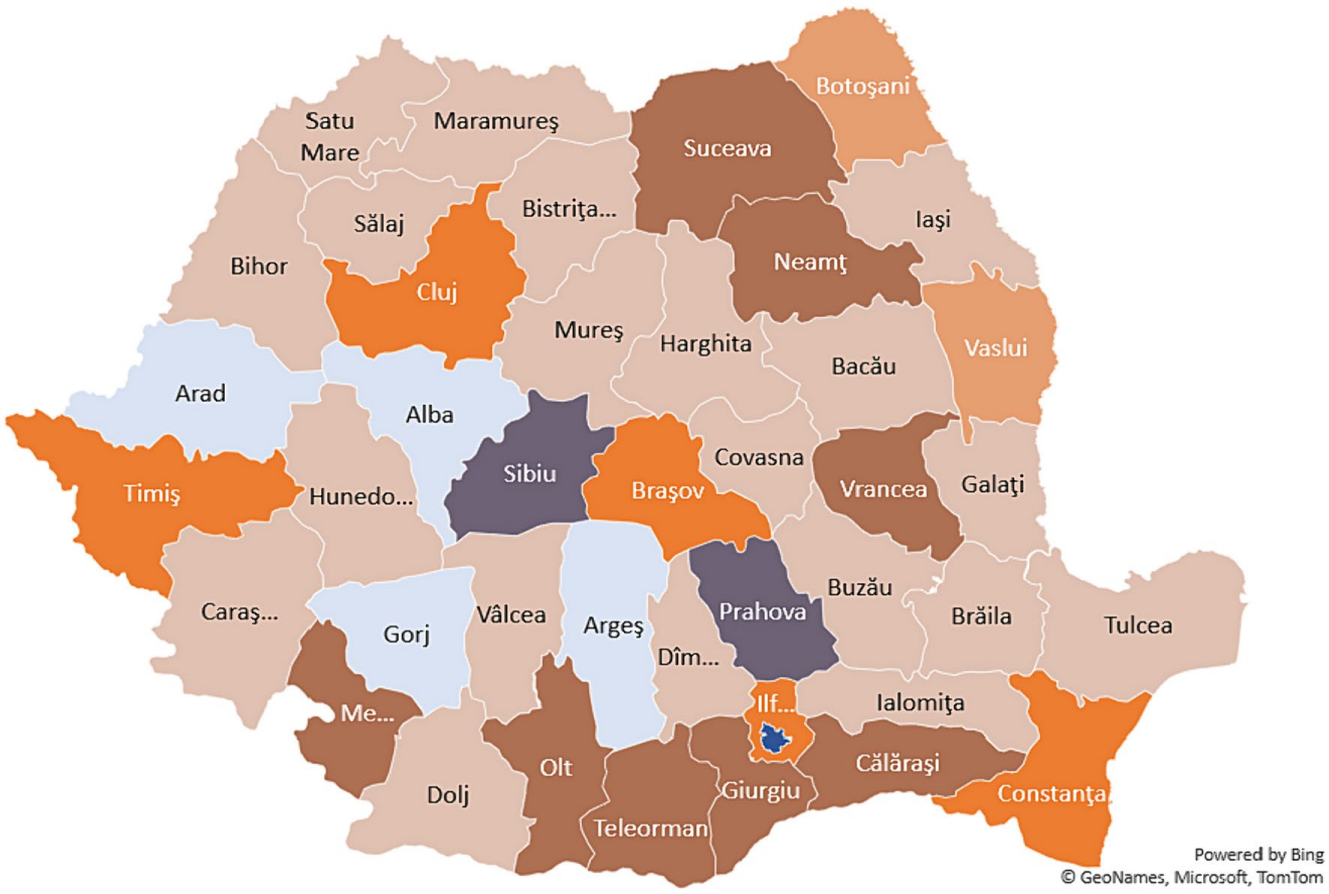
Group of counties based on cluster analysis (Geographical representation). Source: authors’ prelucration based on SPSS26 program.

This section presents the results obtained following a Hierarchical Cluster Analysis based on a number of indicators presented in Table 2. We selected six representative categories that could offer an image on the structural position of each Romanian county: (i) Health (ii) Economic Development (iii) Entrepreneurship (iv) Labor market (v) Local public revenues, and (vi) Local public expenditures (Table 3). All indicators – excepting the COVID-19 impact -, are calculated as average values for the entire 2010–2021 period (see Figure 6).

**Table 2:** Selected categories and indicators for cluster analysis.

Category	Indicator	Source
Health	COVID impact (Death rate of COVID in total cases) Doctors per 1,000 persons	INS
Economic development	GDP per capita	INS
Entrepreneurship	Companies at 1000 persons	INS
Labor market	Employees per 1,000 persons Unemployment rate (the share of unemployed persons in the average number of employees)	INS
Public revenues	Local public revenues	MDPLA
Public expenditures	Investment expenditures (capital spending and EU funds) Human capital expenditures (education and health local public spending) Social protection expenditures Personnel expenditures	MDPLA MDPLA MDPLA MDPLA

Source: authors’ prelucration.

**Table 3:** Descriptive statistics.

Descriptive statistics					
	*N*	Minimum	Maximum	Mean	Std. Deviation
Covid	42	1.00	5.70	3.5119	1.21659
GDPcapita	42	18673.00	102566.00	33723.5952	14161.03731
Companies	42	14.00	76.90	31.5071	13.38095
Empl	42	166.70	615.30	291.6524	91.43983
Unempl	42	1.70	28.00	11.6000	6.05249
Pub_rev	42	2871.00	5780.00	3672.5714	578.87288
Pub_invest	42	688.00	1714.00	1032.3333	228.39319
Pub_humcap	42	662.00	1074.00	806.5000	89.34183
Pub_socialprot	42	121.00	469.00	270.5476	82.58069
Pub_person	42	854.00	1360.00	1106.3810	119.03370
Valid N (listwise)	42				

Source: authors’ prelucration based on Eviews 8 program.

The results of the Hierarchical Cluster Analysis reflect common features among Romanian counties presented in Table 4. The main driver of the clustering process is the economic development, approximated by the GDP per capita, being the most common feature among counties from the same cluster. However, there are also other important characteristics, such as local public revenues or public personnel expenditures that link several groups of Romanian counties.

**Table 4:** Selected categories and indicators for cluster analysis.

Cluster	Counties	Common features
Cluster 1	Alba, Arad, Argeș, Gorj	GDP per capita Local public revenues Local public investment expenditures Local public personnel expenditures
Cluster 2	Bacau, Bihor, Bistrita-Nasaud, Braila, Buzau, Covasna Dambovita, Dolj, Galati, Harghita, Hunedoara, Ialomita, Iasi Maramures, Mures, Satu Mare, Salaj, Tulcea, Valcea	GDP per capita Human capital public expenditures Local public personnel expenditures Employees at 1000 persons
Cluster 3	Botosani, Vaslui	GDP per capita Companies at 1000 persons Employees at 1000 persons Medical staff at 1000 persons Local public revenues Local public investment expenditures Local public personnel expenditures Human capital public expenditures
Cluster 4	Timis, Cluj, Brasov, Ilfov, Constanta	GDP per capita Local social protection expenditures Local public personnel expenditures
Cluster 5	Suceava, Neamt, Vrancea, Olt, Giurgiu, Călărași, Mehedinți, Teleorman	Medical staff at 1000 persons GDP per capita Companies at 1000 persons Employees at 1000 persons Local public revenues Local public personnel expenditures
Cluster 6	Sibiu, Prahova	GDP per capita COVID impact Companies at 1000 persons Employees at 1000 persons Local public revenues Local public personnel expenditures Human capital public expenditures
Cluster 7	Bucuresti	

Source: authors’ prelucration based on SPSS26 program.

## 7 Conclusion and further discussions

This article analyzed the impact of the COVID-19 pandemic among Romanian counties and how the local public authorities have changed the structure of public spending at the local level during the last decade, in particular before and during the COVID-19 pandemic.

Our empirical analysis shows that between 2010 and 2021, the impact of social assistance expenditures was higher than public investment (capital spending and EU funds) on the GDP per capita among Romanian counties. The results of the panel data model at the county level show that a 1% increase in social assistance spending leads to a simultaneous increase in GDP per capita by 0.25 p.p. while an increase in investment spending in the previous period (t-2 years) generates an increase in GDP per capita in the present with 0.18 p.p. Thus, we emphasize that for the majority of Romanian counties, the role of social assistance expenditures is essential for the local welfare and to support individuals.

Furthermore, we presented that in 2020 – at the peak of COVID-19 consecutive waves -, there were counties that significantly increased their investment expenditures to support local economies in terms of GDP. Moreover, the results of our analysis show that education, healthcare, and social assistance expenditures decreased significantly in some counties where the COVID-19 impact was very high in terms of number of deaths (Brăila, Argeș and Suceava).

Last but not least, based on various macroeconomic and structural indicators, we generated seven clusters of counties based on their structural characteristics (health, labor market, economic development, entrepreneurship, and both local public total revenues and several types of public expenditures). Thus, the clusters resulted based on our analysis are:

- Cluster 1 – Alba, Arad, Arges, Gorj;
- Cluster 2 – Bacau, Bihor, Bistrita-Nasaud, Braila, Buzau, Covasna Dambovita, Dolj, Galati, Harghita, Hunedoara, Ialomita, Iasi Maramures, Mures, Satu Mare, Salaj, Tulcea, Valcea;
- Cluster 3 – Botosani, Vaslui;
- Cluster 4 – Timis, Cluj, Brasov, Ilfov, Constanta;
- Cluster 5 – Suceava, Neamt, Vrancea, Olt, Giurgiu, Călărași, Mehedinți, Teleorman;
- Cluster 6 – Sibiu and Prahova;
- Cluster 7 – Bucuresti.

The general tendency highlights that the level of economic development (approximated by the GDP per capita) is the most common feature among counties from the same cluster. However, there are also other important characteristics, such as local public revenues or public personnel expenditures.

In terms of policy-making, the results obtained are relevant for the central government and local public administration to identify the best intervention instruments to support the aggregate demand amid economic shocks, but also to understand some factors that characterize the most exposed counties. As we show, there were counties where the expenditures patterns from 2010 to 2021 contributed to the COVID-19 negative impact but also the interventions in 2020 accentuated the COVID-19 effects (for example, the reduction of healthcare and education expenses).

Further directions of research shouldfocus in our view around two main drivers. Firstly, the mechanism of how social assistance expenditures contribute to local (county) GDP per capita, as our results show that investment expenditures, including EU funds and capital spending - which, traditionally, are considered more important to growth (28) – had a limited contribution to the GDP per capita growth rate.

Secondly, another research domain could analyze more the GDP change during COVID-19 shock, both in counties with intensive economic activity (industry-oriented, rich counties) and counties with low economic activity and standard of living. For example, these were the cases for Arges and Dambovita, or Timis and Botosani, where the structure of local economies is very different, but between 2019 and2020 the GDP contraction was quite similar.

## Data availability statement

The original contributions presented in the study are included in the article/Supplementary material, further inquiries can be directed to the corresponding author.

## Author contributions

GȘ, DB, and AA contributed to the conception and design of the study. GȘ, AdP, and AnP organized the database. GȘ, AH, AnP, and AA performed the statistical analysis, econometric techniques, and results interpretation. DB, GȘ, AnP, and AdP wrote the first draft of the manuscript. DV, AH, and AdP wrote sections of the manuscript. All authors contributed to manuscript revision, read, and approved the submitted version.

## Footnotes

^1^http://www.dpfbl.mdrap.ro/sit_ven_si_chelt_uat.html, accessed in November 2022.

^2^http://statistici.insse.ro:8077/tempo-online/, accessed in November 2022.

^3^https://cnp.ro/prognoze/?lang=en, accessed in November 2022.

^4^2010–2021, respectively 2013–2021 after the adjustments of the Eviews 8 program.

^5^Overall, the richest five counties Bucharest, Cluj, Timisoara, Constanta, and Iasi represents 50% of the Romanian GDP.
