# Supplementary material for: The intervention of local public authorities and the impact of the COVID-19 pandemic in Romania: a subnational analysis
Source: Front Public Health. 2024 May 16;12:1105518. doi: 10.3389/fpubh.2024.1105518 (PMC11141162; doi:10.3389/fpubh.2024.1105518)
Supplement: Supplementary file 1 [file Data_Sheet_1.docx]

**Annexes**


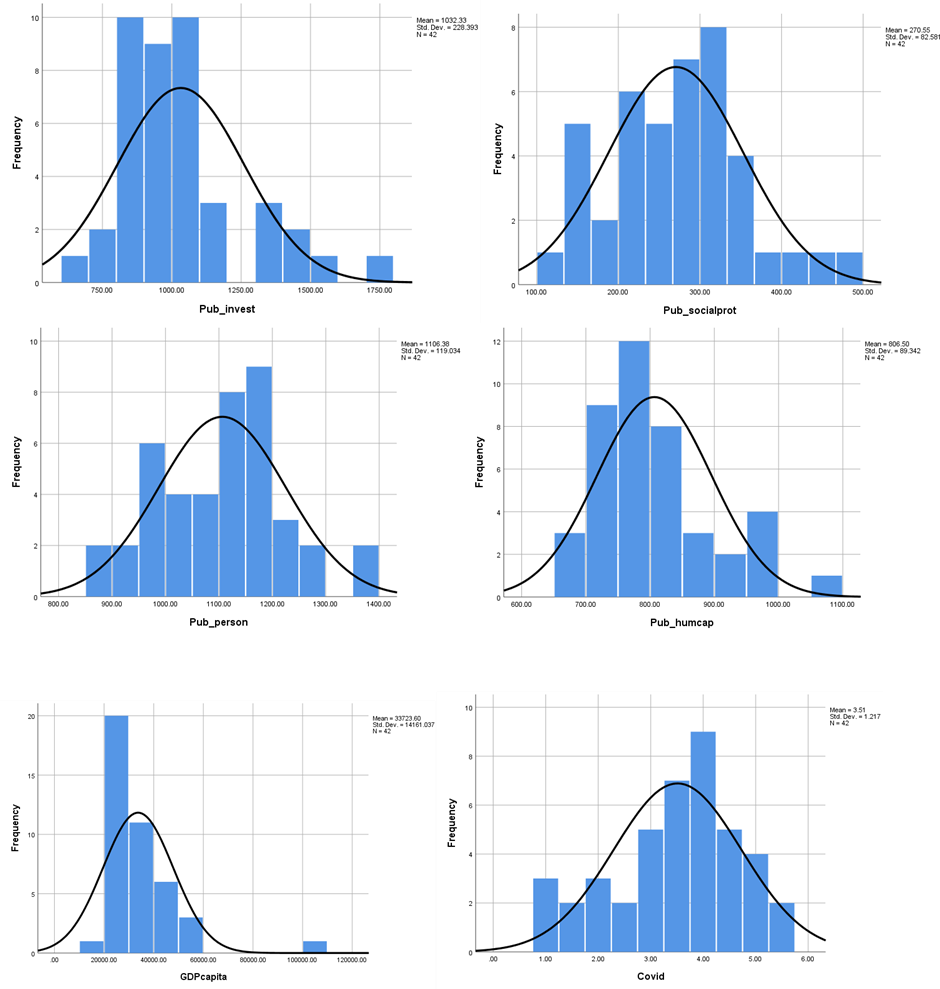


Source: authors', based on SPSS26 program

**COVID-19 impact at county level (% of COVID-19 death in the total number of cases)**


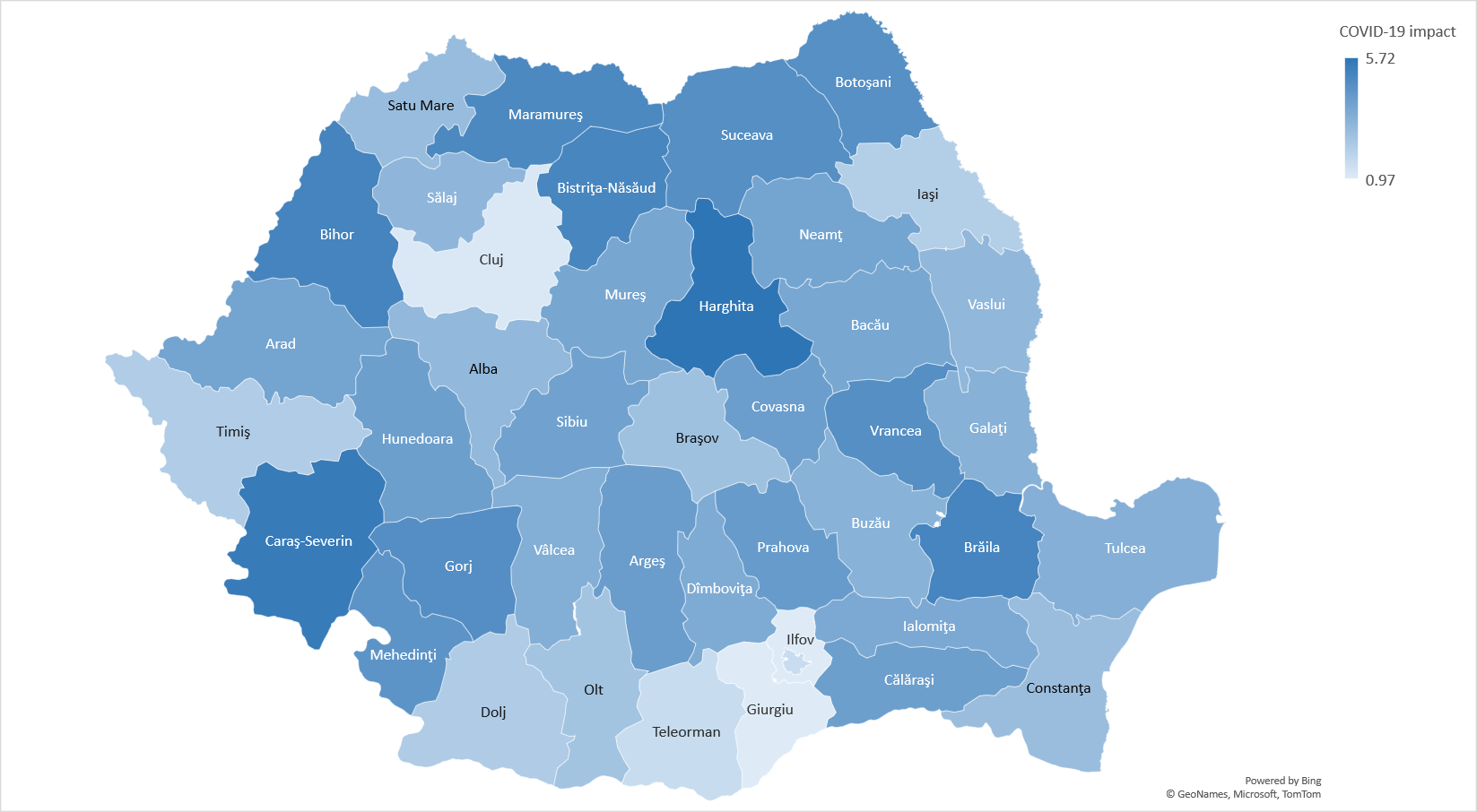


Source: authors', based on data from the National Committee for the coordination of vaccination against COVID-19 and the National Institute for Public Healthcare
